# Lessons Learned From AI-Assisted Guideline Generation in Parastomal Hernia Repair

**DOI:** 10.3389/jaws.2026.15992

**Published:** 2026-04-21

**Authors:** Maciej Śmietański, Mateusz Zamkowski, Krzysztof Tyszka

**Affiliations:** 1 Śmietański Hernia Center, Lux Med Hospital in Gdańsk, Gdańsk, Poland; 2 II Department of Radiology, Medical University of Gdańsk, Gdańsk, Poland; 3 Institute of Experimental Physics, Faculty of Physics, University of Warsaw, Warsaw, Poland

**Keywords:** Artificial intelligence, consensus, EHS guidelines, GPT-5, parastomal hernia

## Abstract

**Background:**

Large language models (LLMs) can analyse scientific literature and draft medical recommendations, but their role in formal clinical guideline development is unclear.

**Aim:**

To evaluate whether a publicly available GPT-based LLM can generate coherent, GRADE-based guidelines for parastomal hernia management from a predefined evidence base, and to compare these with the 2017 European Hernia Society (EHS) guidelines. A secondary aim was to explore implications for academic publishing and scientific authorship.

**Materials and Methods:**

The 2017 EHS parastomal hernia guidelines (Antoniou et al.) were used as the reference framework. Within a closed session, the model was instructed to apply AGREE II and GRADE principles to 52 full-text clinical papers mirroring the original EHS reference set, and to formulate recommendations for nine key clinical questions (KQs). For each KQ, the model defined PICO, summarized the evidence, rated certainty, and stated direction and strength of recommendation. AI-derived guidance was then systematically compared with EHS statements. Divergences were classified as interpretative, threshold-based (handling of low-certainty evidence), or evidence-weighting.

**Results:**

AI-generated recommendations showed full or near-full alignment with EHS guidance in most domains, including diagnosis, prophylactic mesh for permanent end colostomy, rejection of suture-only repair, preference for non-keyhole laparoscopic repair, and favouring synthetic over biologic meshes. Differences arose primarily where evidence was very low quality: the model issued cautious, conditional recommendations (e.g., watchful waiting in asymptomatic hernias, consideration of laparoscopy in suitable patients, preference for retromuscular synthetic mesh and avoidance of cross-linked collagen onlay), whereas EHS opted for no recommendation.

**Conclusion:**

Within a closed evidence base, a GPT-based model can reproduce the logic and structure of expert guideline development with high fidelity. Discrepancies mainly reflect different thresholds for acting on low-certainty evidence, supporting a complementary role for AI as a structured methodological and drafting assistant rather than a replacement for human consensus.

## Introduction

Artificial intelligence (AI) has rapidly evolved from a theoretical computational concept into a practical and indispensable medical tool. Its use spans diagnostic imaging, decision-support systems, outcome prediction, and treatment planning. Recent advancements in large language models (LLMs), such as the GPT (Generative Pre-trained Transformer) architecture, have introduced the possibility of interpreting scientific literature, performing reasoning tasks, and drafting guideline-like recommendations [1–3].

Beyond its clinical applications, AI is increasingly employed in the analytical and academic dimensions of medicine. Large language models are capable of reviewing, summarizing, and even structuring scientific literature for systematic reviews and meta-analyses. Furthermore, AI has shown strong potential in research design, including statistical planning and cohort definition for prospective clinical trials [4, 5]. Consensus groups and scientific societies are also beginning to use AI to support evidence synthesis for their guideline development processes [6]. This growing presence of AI in scientific research has prompted an urgent need to define the ethical and methodological boundaries of its use. On one hand, the computational capacity of generative intelligence surpasses any human’s ability to process and correlate data. On the other, questions arise about how AI systems evaluate evidence when confronted with false, incomplete, or biased publications. Such flaws can directly influence the validity of AI-derived conclusions.

Academic institutions and scientific publishers are actively working to define these boundaries. What was considered unacceptable only a few years ago—such as openly using machine learning models for translation or text editing—is now common practice [7]. Nevertheless, most peer-reviewed journals maintain strict limits on AI use in manuscript authorship, requiring transparent disclosure of the model’s role.

A further limitation arises from the commercial architecture of the global publishing market. Many scientific journals restrict access to their content, preventing open AI systems from using full-text data for analysis. This closed-access paradigm, designed to protect commercial interests, effectively limits the potential of AI to engage with comprehensive, up-to-date scientific evidence. A shift toward open data availability could not only disrupt traditional publishing economics but also profoundly reshape how scientific achievements are produced, assessed, and disseminated.

The authors of the present study, as active participants in international consensus panels (including European Hernia Society – EHS, European Association for Endoscopic Surgery - EAES and International Endo-Hernia Society - IEHS) and co-authors of multiple surgical guidelines, aim to explore this frontier. Specifically, we sought to determine whether an AI model could independently generate coherent clinical guidelines when provided with complete scientific data, and how its conclusions would compare with expert-derived recommendations. The case of parastomal hernia repair, an area with well-structured but complex evidence, was chosen as a representative example of guideline formulation in modern hernia surgery.

### Aim

The primary objective of this study was to determine whether publicly available AI models can generate coherent medical guidelines for parastomal hernia repair using existing full-text data. The AI-derived conclusions were compared with the official EHS guidelines for congruency [8].

A secondary objective was to evaluate the potential influence of AI-driven guideline development on the scientific publishing ecosystem and academic career structures.

## Materials and Methods

### Study Framework

The European Hernia Society (EHS) guidelines on the prevention and treatment of parastomal hernias were used as the baseline framework. GPT-5.1 (OpenAI, San Francisco, USA) was instructed to:

1. Retrieve methodological frameworks for clinical guideline development (AGREE II, GRADE).
2. Review and analyze 52 full-text papers corresponding to the original EHS reference database.
3. Generate structured recommendations addressing nine key clinical questions adopted from the EHS consensus.

### Data Sources and Evidence Base

To approximate the evidence base used by the EHS guideline group, the authors compiled a set of 52 full-text clinical articles on parastomal hernia, covering:

– Definitions and diagnostic criteria (clinical examination, CT, ultrasound),
– Incidence and risk factors,
– Classification systems (clinical, radiological, and EHS types),
– Prophylactic mesh implantation at the time of stoma formation,
– Elective repair techniques (open and laparoscopic),
– Mesh positions and mesh materials (synthetic, biologic, 3D/funnel meshes),
– And, where available, cost-effectiveness analyses.

In addition, two key methodological papers were included: the AGREE II instrument for guideline appraisal and the GRADE framework for rating certainty of evidence and formulating recommendations. These documents were provided to the model to standardize its understanding of guideline methodology.

All articles were uploaded in full-text PDF form within a single AI session. The model was explicitly instructed to base its recommendations only on these uploaded documents and the EHS guideline text, and to refrain from introducing additional external references or data.

### AI Model and Prompting Strategy

A GPT-based large language model (OpenAI, San Francisco, USA) was accessed via a conversational interface. The interaction followed a structured protocol defined *a priori* by the authors:

1. The model was informed that the task was to simulate the development of clinical guidelines for parastomal hernia, using AGREE II and GRADE principles.
2. The EHS 2017 guideline document was introduced as the reference framework, but the model was explicitly asked not to simply replicate its conclusions.
3. The set of 54 uploaded documents (52 clinical articles and 2 methodological papers) was then uploaded in batches, and the model was prompted to internally structure the information by study design, population, interventions, outcomes, and follow-up).
4. The model was instructed to generate recommendations exclusively on the basis of these uploaded papers, using the GRADE methodology (explicit PICO, summary of evidence, certainty of evidence, and direction/strength of recommendation).

All prompts were issued in Polish, reflecting the native language of the investigators; the AI outputs were generated in English (Supplementary Material 1). The model was allowed to ask for clarification only if necessary, but was not permitted to call external web resources or databases. The full conversation was exported and archived as the raw AI output for analysis.

### Key Clinical Questions (as Defined by EHS 2017)

To ensure comparability with the EHS guideline, the authors adopted the nine key clinical questions (KQs) used in the original 2017 document:

1. What is the diagnostic accuracy of clinical examination versus imaging in parastomal hernia detection?
2. Is there a role for watchful waiting in parastomal hernia management?
3. Are there stoma creation techniques without mesh that reduce parastomal hernia incidence?
4. Does prophylactic mesh placement during stoma construction reduce hernia occurrence?
5. Is suture repair an acceptable option for elective parastomal hernia repair?
6. Is laparoscopic repair equivalent to open repair in elective cases?
7. What is the optimal open repair technique?
8. What is the optimal laparoscopic repair technique?
9. Which mesh types are most effective?

For each KQ, the model was asked to:

1. Define the relevant PICO,
2. Summarize the evidence from the uploaded papers,
3. Rate the certainty of evidence according to GRADE categories (high, moderate, low, very low), and
4. Formulate a recommendation with specified direction (for/against/no recommendation) and strength (strong/weak).

### Comparison With EHS Recommendations

The EHS 2017 recommendations corresponding to the nine KQs were extracted from the original guideline document. Two authors (hernia surgeons involved in guideline work) independently compared, for each KQ:

– The direction of the recommendation (for/against/no recommendation),
– the strength (strong/weak), and
– The stated certainty or quality of evidence.

For each domain, the AI-derived recommendation was classified as:

– Full alignment (same direction and similar strength),
– Partial alignment (same general direction but different strength or explicitness), or
– Divergence (different direction or clearly different clinical strategy).

Discrepancies were further categorized as:

– Interpretative – related to wording, framing, or minor linguistic/contextual nuances;
– Threshold-based – arising from different handling of low-certainty evidence (e.g., the model issuing a weak, conditional recommendation where EHS chose “no recommendation” despite observing the same signal);
– Evidence-weighting – where the probabilistic model appeared to place disproportionate weight on a small number of negative or positive studies compared with the human panel. In our material, this was most visible in explicitly negative recommendations generated by the model (e.g., against specific mesh types), which were consistently linked to at least one publication reporting poor outcomes, whereas the EHS panel opted to remain neutral or non-committal. These discrepancies likely reflect how the model, given its training data, prompt and safety/alignment constraints, translated sparse adverse signals into cautious negative wording, rather than any deterministic algorithm or explicit rule that would systematically override the guideline panel’s judgement.

Any disagreements in classification between the two reviewers were resolved by discussion and consensus. No formal statistical testing was performed, as the aim of the study was descriptive and exploratory rather than hypothesis-driven.

## Results

AI-generated recommendations demonstrated a high degree of consistency with the EHS guideline framework, showing full or near-full alignment in six of nine analyzed domains. In most instances, the model reproduced the logical structure, evidence hierarchy, and direction of recommendations formulated by the EHS expert panel.

In the area of diagnosis, both EHS and the AI model emphasized clinical examination in supine and erect positions with Valsalva manoeuvre as the primary diagnostic tool, with cross-sectional imaging (CT or 3D intrastomal ultrasound) reserved for equivocal cases or preoperative planning. The model tended to rate the certainty of evidence as “low”, while the EHS panel frequently classified the same data as “very low”, but the clinical conclusions remained essentially identical.

There was complete agreement regarding prophylactic mesh for elective permanent end colostomy, discouraging suture-only repair, and favouring a mesh without a central keyhole (modified Sugarbaker configuration) in laparoscopic repair. Both sources also converged on the general preference for synthetic, non-absorbable lightweight meshes over biologic materials, particularly in intraperitoneal positions.

Divergences emerged primarily in areas where the underlying data are sparse and of very low quality. For watchful waiting in asymptomatic or minimally symptomatic parastomal hernias, the EHS guidelines chose not to issue a formal recommendation, whereas the AI model formulated a weak recommendation in favour of observation, based on the combination of high recurrence rates after repair and the observation that many radiological hernias never become symptomatic.

For the operative approach (laparoscopic vs. open), the EHS panel again remained neutral, citing heterogeneity of available studies. The AI model interpreted registry and cohort data as supporting a conditional preference for laparoscopy in suitable patients and experienced centres, based on shorter length of stay and lower wound morbidity, while acknowledging low certainty of evidence.

Similarly, for open repair technique and mesh type, EHS guidelines refrained from specifying an optimal configuration, whereas the AI model suggested that, when technically feasible, a retromuscular/preperitoneal synthetic mesh could be preferred and issued a strong negative opinion on cross-linked collagen onlay repair, which showed very high recurrence rates in a long-term series.

Overall, discrepancies were driven not by conflicting interpretation of the same data, but by the model’s tendency to translate consistent observational signals into conditional recommendations, whereas the EHS panel more often chose a conservative “no recommendation” stance when evidence was graded as very low. A detailed domain-by-domain comparison is presented in Table 1.

**TABLE 1:** Comparison of EHS 2017 recommendations and GPT-5.1-generated recommendations for parastomal hernia management.

Domain	EHS 2017 recommendation	GPT-5.1-derived recommendation	Alignment	Comment
Diagnosis	Clinical examination in supine and erect positions with Valsalva is essential; CT/US may be used in uncertain cases	Clinical examination as the primary diagnostic modality; CT (preferably with a dedicated protocol) or 3D intrastomal ultrasound suggested when findings are equivocal or for operative planning	Full	Both emphasize clinical exam as first-line and imaging as an adjunct; GPT-5.1 is slightly more explicit about when to use imaging, but the clinical strategy is identical
Watchful waiting	No formal recommendation due to insufficient comparative evidence	Weak recommendation supporting watchful waiting in asymptomatic or minimally symptomatic parastomal hernias, with surgery reserved for symptomatic or complicated cases	Partial	EHS adopts a neutral stance; GPT-5.1 converts observational patterns (high recurrence after repair vs. many stable hernias) into a conditional recommendation
Stoma construction (route/plane)	No preference between extraperitoneal vs. transperitoneal route; no recommendation for transrectus vs. pararectus; suggestion to keep fascial aperture as small as possible	No specific recommendation for route or muscle plane; suggestion to avoid unnecessarily large fascial apertures and to optimize modifiable risk factors (e.g., BMI, waist circumference)	Full	Both sources refrain from favouring a particular non-mesh technique and emphasize the importance of aperture size; GPT-5.1 additionally highlights risk-factor optimization
Prophylactic mesh	Strong recommendation for prophylactic synthetic non-absorbable mesh in elective permanent end colostomy; no recommendation for other stoma types or for biologic meshes	Strong recommendation for prophylactic lightweight synthetic mesh in elective permanent end colostomy; suggestion against routine use of biologic meshes for prophylaxis	Full for indication; partial for biologics	The indication for synthetic mesh in end colostomy is identical. GPT-5.1 goes one step further by formulating a negative conditional recommendation for biologic meshes, whereas EHS remains neutral
Suture-only repair	Suture-only repair discouraged in elective parastomal hernia surgery	Suture-only repair discouraged in elective parastomal hernia surgery; mesh repair recommended whenever feasible	Full	Complete concordance in direction and strength of recommendation
Operative approach (laparoscopic vs. open)	No recommendation in favour of laparoscopic or open repair due to heterogeneous and low-quality evidence	Weak recommendation suggesting consideration of laparoscopy in suitable patients and experienced centres, based on shorter length of stay and lower wound morbidity	Partial	GPT-5.1 translates observational data into a conditional preference for laparoscopy; EHS opts for formal neutrality
Laparoscopic repair method	Mesh without a central keyhole (e.g., modified Sugarbaker) suggested over keyhole mesh	Mesh without a central keyhole (modified Sugarbaker) identified as preferred technique; keyhole repair not recommended as standard due to higher recurrence	Full	Both favour a non-keyhole configuration; GPT-5.1 expresses a clearer negative stance towards pure keyhole techniques
Open repair technique	Insufficient evidence to recommend a specific open mesh position; no recommendation for or against biologic onlay	Mesh-based open repair recommended over suture or relocation; retromuscular/preperitoneal synthetic mesh suggested when feasible; strong recommendation against cross-linked collagen onlay due to high recurrence	Partial	EHS remains non-committal; GPT-5.1 proposes a preferred plane (sublay) and explicitly discourages cross-linked collagen onlay based on poor long-term outcomes
Mesh material	No evidence for superiority of biologic over synthetic mesh; no specific recommendation on material	Synthetic, non-absorbable lightweight meshes recommended for prophylaxis and repair; routine use of biologic/cross-linked collagen meshes (especially onlay) discouraged	Partial	Both recognize lack of advantage of biologics; GPT-5.1 formulates this as an explicit preference for synthetic meshes and a negative recommendation for biologic onlay, especially when high recurrence has been documented

## Discussion

This study examined whether a GPT-based large language model, when restricted to a predefined set of clinical papers and guided by established guideline methodology, could generate recommendations for parastomal hernia management that resemble those of the 2017 European Hernia Society (EHS) guidelines. Within this constrained framework, the model proved capable of reproducing not only the overall direction of the EHS recommendations, but also much of their internal logic, structure and use of the GRADE framework. The key differences between human and AI-derived guidance emerged predominantly in areas where the underlying evidence is sparse, heterogeneous and of very low quality, and they reflected distinct approaches to translating such evidence into formal recommendations rather than fundamentally divergent interpretations of the data.

### Areas of Concordance

In domains where the evidence base is relatively robust, the alignment between the AI-generated guidance and the EHS consensus was striking. Both approaches emphasized clinical examination in supine and erect positions with Valsalva manoeuvre as the primary diagnostic tool for parastomal hernia, with cross-sectional imaging reserved for specific indications. The model, mirroring the EHS guideline, consistently treated CT and advanced ultrasound techniques as adjuncts rather than replacements for clinical evaluation. Imaging was suggested in situations where the clinical picture was equivocal, where a more detailed anatomical assessment was needed prior to operative repair, or when differentiating between bulging, stomal prolapse and a true parastomal hernia was clinically relevant. Although the model tended to label the certainty of evidence for these diagnostic recommendations as “low” rather than “very low,” the practical implications remained essentially identical to those of the EHS document.

The degree of concordance was equally high in the preventive and therapeutic domains in which randomized trials and meta-analyses are available. Both the AI-derived guideline and the EHS recommendations endorsed the use of prophylactic synthetic mesh at the time of elective permanent end colostomy, noting that multiple trials have demonstrated a meaningful reduction in parastomal hernia incidence without a significant increase in mesh-related morbidity. In the treatment setting, both sources issued a strong negative recommendation against suture-only repair in elective surgery, recognising the consistently high recurrence rates observed after fascial repair alone. Likewise, the model reproduced the EHS panel’s preference for a mesh configuration without a central keyhole in laparoscopic repair—functionally corresponding to the modified Sugarbaker technique—because of its lower recurrence rates compared with keyhole repairs. Finally, both approaches implicitly converged on synthetic, non-absorbable lightweight meshes as the default materials for prophylaxis and repair, acknowledging that biologic meshes have not demonstrated superiority and may in some contexts be associated with poorer durability.

These areas of agreement suggest that, when provided with the same randomized and observational evidence and with explicit prompts to apply AGREE II and GRADE concepts, a large language model can reconstruct much of what a human guideline panel does: identify clinically important outcomes, weigh benefits and harms, and translate them into structured recommendations with stated certainty. Importantly, this convergence was not limited to “trivial” questions; it also encompassed nuanced trade-offs, such as accepting intraperitoneal mesh implantation around a stoma in exchange for a reduction in long-term hernia risk, provided that infection rates remain low and patient survival justifies prophylaxis.

### Areas of Divergence and Their Origin

The most interesting findings of this study, however, lie in the domains where the model and the EHS panel diverged. These discrepancies did not stem from opposing interpretations of individual trials but rather from different attitudes toward low-certainty evidence. When faced with consistent but observational signals—such as high recurrence rates after repair or modest but reproducible differences in postoperative recovery between operative approaches—the AI system tended to formulate weak, conditional recommendations, whereas the human panel often concluded that “no recommendation” could be made.

Watchful waiting in asymptomatic or minimally symptomatic parastomal hernia is a clear example of this phenomenon. The EHS guideline group explicitly stated that the available evidence did not allow a recommendation for or against conservative management. By contrast, the AI model, given the same data, proposed a weak recommendation in favour of observation for patients without significant symptoms, suggesting that surgery should be reserved for those with pain, appliance problems, obstructive episodes or major skin complications. The model’s rationale was straightforward and internally consistent: many radiologically detected parastomal hernias never progress to clinically relevant symptoms, whereas surgical repair—particularly when performed without mesh or with relocation alone—is associated with a high risk of recurrence and non-trivial perioperative morbidity. From a strictly GRADE-based perspective, one may argue that the model overstepped by turning such patterns into guidance; nevertheless, its reasoning is transparent and mirrors the intuitive risk–benefit considerations that many clinicians apply in practice.

A similar pattern was observed in the comparison between laparoscopic and open repair. The EHS panel refrained from endorsing either approach, citing heterogeneity and methodological limitations of the available studies. In contrast, the AI-derived guideline interpreted registry analyses and retrospective series as supporting a cautious preference for laparoscopic repair in appropriately selected patients and experienced centres, mainly on the basis of shorter length of stay and lower rates of wound complications. The model explicitly labelled the certainty of evidence as low and did not claim superiority in terms of recurrence, but it took the additional step of translating these advantages into a weak recommendation. Again, the underlying data are not in dispute; what differs is the threshold at which signals from imperfect evidence are deemed sufficient to justify explicit guidance.

The specification of open repair technique and mesh material offers a third example. The EHS guidelines describe the evidence regarding optimal mesh position (onlay, sublay, intraperitoneal) and mesh type (synthetic vs. biologic, PVDF vs. composite) as insufficient for firm conclusions, and they avoid recommending one configuration over another. The AI model, by contrast, proposed that a retromuscular or preperitoneal synthetic mesh could be preferred when technically feasible, drawing on parastomal series with favourable outcomes and by analogy with broader ventral hernia literature. It also expressed a strong negative view of cross-linked collagen onlay repair, which in one long-term series demonstrated very high recurrence rates. From a conservative guideline perspective, basing a specific “against” recommendation on a single series is arguably too assertive; nonetheless, the logic is again deterministic: if one technique repeatedly fails in the available data and no counterevidence exists, a recommendation to avoid it can be defended as a cautious interpretation of harm.

Taken together, these examples suggest that the model may appear more willing than human panels to translate consistent but very low-certainty observational signals into conditional recommendations. This should not be interpreted as superior judgment. Rather, it reflects a structural difference between probabilistic pattern recognition and formal guideline development under GRADE, where the strength of recommendation must remain explicitly separated from certainty of evidence and must also incorporate normative judgments about acceptable uncertainty, patient preferences, feasibility, equity, and resource implications. In this sense, the present findings reinforce—not weaken—the importance of human consensus in guideline development.

### Potential Implications for Future

The potential implications for future guideline processes are multifaceted. On the one hand, the high degree of equalization in key domains demonstrates that large language models can already function as valuable methodological tools: they are capable of rapidly summarizing large evidence bases into PICO-structured synopses, of making the chain of reasoning behind recommendations explicit, and of highlighting domains in which data are weak but internally consistent. This could help guideline panels focus their discussions on areas where human judgment is genuinely required, such as weighing patient preferences, resource constraints and implementation feasibility, rather than on manually collating and restating data that AI can process efficiently.

On the other hand, the divergences observed in this study underscore that AI systems do not and cannot replace the normative components of guideline work. They do not possess values, do not understand local healthcare systems, and do not bear the consequences of over- or under-treatment. Their readiness to issue weak recommendations on the basis of fragile evidence may be useful as a catalyst for expert debate, but it is not a substitute for deliberation about what degree of uncertainty is acceptable in different clinical and societal contexts. For the foreseeable future, AI-generated guidelines should therefore be viewed as structured proposals or “first drafts” to be interrogated, refined and, where necessary, rejected by human experts, rather than as standalone directives.

### Study Strengths and Limitations

Strengths and limitations of this study should be acknowledged. A major strength is that the experiment was explicitly anchored in internationally accepted methodological frameworks for guideline development, namely, AGREE II and GRADE. A second strength is the restriction of the model to a closed evidence base designed to mirror the literature underpinning the 2017 EHS guideline, which reduced the risk of trivial concordance and strengthened the internal validity of the comparison. A third strength is the transparent, domain-by-domain comparison between AI-derived and EHS recommendations.

This study also has important limitations. First, although the model was able to summarize studies and formulate GRADE-like recommendations, its evidence assessment remains fundamentally different from that of human guideline panels. In particular, it cannot meaningfully evaluate key normative GRADE domains such as patient values and preferences, acceptability, feasibility, equity, and resource use. Second, the evidence base was preselected rather than derived through a *de novo* systematic review, which limits generalizability and introduces a potential risk of selection bias. Third, the comparison with the EHS recommendations relied on expert judgment by two authors and did not include formal inter-rater reliability metrics. Finally, the experiment was performed using a single model version and a single conversational run; different models, versions, or prompting strategies may produce different outputs. In addition, although the model was instructed to rely exclusively on the uploaded material, complete isolation from prior training data cannot be guaranteed.

### Implications for Academic Structures

The integration of AI into scientific workflows will inevitably require redefinition of academic career metrics. If AI can autonomously generate systematic reviews, meta-analyses, or consensus drafts, the traditional evaluation of individual authorship may become obsolete. Prompt design, critical interpretation, and validation of AI output should emerge as new academic competencies, akin to the adoption of biostatistics in the 20th century.

### Future of Real-Time Guideline Updates

Traditional guideline cycles—typically 3–5 years—create latency in knowledge translation. The ability to update guidelines in real time, synchronized with new data publication, could dramatically accelerate evidence dissemination. However, this would require open databases, standardized metadata, and transparent algorithmic oversight.

### Ethical and Philosophical Dimensions

AI should be viewed as an advanced methodological tool that may support evidence synthesis, structuring of recommendations, and identification of areas requiring expert discussion. However, its outputs remain dependent on human supervision, critical appraisal, and ethical governance.

## Conclusion

Publicly available large language models can reproduce several analytical components of formal guideline development with high fidelity when constrained to a closed evidence base and explicit methodological frameworks. The main discrepancies observed in this study arose in areas of very low-certainty evidence, where the model tended to formulate conditional recommendations in situations in which the EHS panel chose to remain neutral. These findings suggest that AI may be useful as a structured methodological tool for evidence organization, drafting, and identification of discussion points, but not as a substitute for the normative, contextual, and accountable decision-making that defines GRADE-based guideline development. Final recommendations, authorship, and responsibility remain inseparable from human expert consensus.

## Data Availability

The datasets presented in this study can be found in online repositories. The names of the repository/repositories and accession number(s) can be found in the article/Supplementary Material.
