# Supplementary material for: Lessons Learned From AI-Assisted Guideline Generation in Parastomal Hernia Repair
Source: J Abdom Wall Surg. 2026 Apr 21;5:15992. doi: 10.3389/jaws.2026.15992 (PMC13139038; doi:10.3389/jaws.2026.15992)
Supplement: Supplementary file 1 [file Supplementaryfile1.docx]

User: In a moment I will upload 52 scientific papers on parastomal hernias. I would like you to analyze them, remember them, and then, based only on the information from these papers, answer the Key Questions that I will ask. These will be guidelines concerning the management of parastomal hernias – please prepare the answers using the GRADE system.

Assistant: Great, I understand the plan and it’s a very sensible idea.

What I will do once you upload the 52 papers:

1. Go through all the papers

- identify: study design (RCT, cohort, registry, review, etc.),

- population (type of stoma, time from creation, indications),

- interventions (type of repair, mesh, prophylaxis vs treatment, onlay/retromuscular/laparoscopic, etc.),

- main endpoints (recurrence, complications, quality of life, mortality, reoperations),

- length of follow‑up.

2. Build an internal “database” of these papers

So that when answering your Key Questions I can rely **exclusively** on those 52 publications (according to your request: only information from those papers).

3. Prepare a GRADE framework for each answer:

- PICO for each KQ,

- certainty of evidence: very low / low / moderate / high,

- GRADE domains:

- risk of bias

- inconsistency

- indirectness

- imprecision

- publication bias (if assessable),

- then strength of recommendation:

- strong vs conditional (weak),

- for vs against.

4. Answering the Key Questions

Once you give the specific KQs, I’ll answer in a structure like:

KQ1. [Question text]

- PICO

- Summary of evidence from those 52 papers

- GRADE – certainty of evidence + short rationale

- Recommendation (direction + strength)

- Practical remarks, if clearly supported by the data from those papers.

Important assumptions (as per your request):

- I will **not** rely on any external knowledge beyond what is in these 52 papers (no EHS guidelines, no other literature, no personal/clinical “experience”).

- If a KQ cannot be answered in a meaningful way based only on those papers, I’ll explicitly say there is an evidence gap.

You can upload all 52 files in one or several batches in this same conversation. When you’re done, just tell me that’s all of them and then you can start providing Key Questions (KQ1, KQ2, etc.). I’ll then answer them directly in the same response.

---

[Several rounds follow where the user uploads PDF files. The assistant reads and summarizes them, building an internal evidence base about parastomal hernia – definitions, risk factors, classification systems, diagnostic accuracy of clinical exam vs CT and ultrasound, prophylactic meshes, techniques for stoma creation, open and laparoscopic repair techniques, mesh materials (synthetic, biologic, funnel/3D meshes), recurrence rates, complication profiles, and cost‑effectiveness models. These intermediate steps consist mainly of technical article summaries, already written in English.]

---

User:

1. What is the diagnostic accuracy of clinical examination versus imaging in parastomal hernia detection?

2. Is there a role for watchful waiting in parastomal hernia management?

3. Are there stoma creation techniques without mesh that reduce parastomal hernia incidence?

4. Does prophylactic mesh placement during stoma construction reduce hernia occurrence?

5. Is suture repair an acceptable option for elective parastomal hernia repair?

6. Is laparoscopic repair equivalent to open repair in elective cases?

7. What is the optimal open repair technique?

8. What is the optimal laparoscopic repair technique?

9. Which mesh types are most effective?

Assistant:

Below are answers to your 9 Key Questions, based **only** on the uploaded papers, using a GRADE‑style structure (PICO, certainty, recommendation).

1. What is the diagnostic accuracy of clinical examination versus imaging in parastomal hernia detection?

PICO

Adults with a stoma, assessment for parastomal hernia.

Intervention: clinical examination.

Comparator: imaging (CT, including prone protocol; 3D intrastomal ultrasound).

Outcomes: sensitivity, specificity, inter‑observer agreement, concordance with intraoperative findings.

Summary of evidence

• Inter‑observer reliability of clinical exam is limited. In a cohort of stoma patients, agreement between surgeons on the presence of parastomal hernia was only fair to moderate (kappa ranging roughly 0.29–0.73), indicating that clinical assessment alone is not highly reproducible.

• A study that compared standardized clinical criteria with CT in **prone** position (with a radiopaque ring) found high concordance (kappa ≈ 0.80) between clinical exam and prone CT, whereas standard supine CT was clearly less reliable. This suggests that a carefully standardized clinical exam can correlate well with a high‑quality imaging protocol.

• 3D intrastomal ultrasound shows good inter‑ and intra‑observer reliability (inter‑observer kappa around 0.6–0.7, intra‑observer >0.8) and has a short learning curve (≈30 examinations).

• A direct comparison of 3D ultrasound and CT against intraoperative findings showed that both modalities had high sensitivity (≈83–94%) for detecting parastomal hernia, with 3D US demonstrating higher specificity than CT in that small prospective series.

GRADE – certainty of evidence

• Study sizes are small, definitions of parastomal hernia differ, and reference standards are not always uniform.

• Most data come from single‑centre prospective cohorts, not randomized studies.

→ Certainty: **Low**.

Recommendation

We suggest that a standardized clinical examination (supine and erect, with straining) can often be sufficient to detect clinically relevant parastomal hernias, especially when local practice has been calibrated against high‑quality imaging. However, cross‑sectional imaging (preferably CT with a dedicated prone protocol or 3D intrastomal ultrasound) should be used:

• when clinical findings are equivocal,

• before operative planning,

• when differentiating bulging/protrusion from a true hernia is important, or

• when complications (incarceration, obstruction, fistula) are suspected.

Direction & strength: *For using imaging as an adjunct to clinical examination in selected situations*; **conditional recommendation, low‑certainty evidence**.

---

2. Is there a role for watchful waiting in parastomal hernia management?

PICO

Adults with parastomal hernia; watchful waiting versus elective surgical repair.

Outcomes: progression of symptoms, need for surgery, emergency complications, and operative recurrence/complication rates.

Summary of evidence

• Observational cohorts (CT‑based and clinical) report radiologic parastomal hernia rates of 30–60%, but only a subset of patients go on to require operative repair. In one series, roughly a third of patients with parastomal hernia underwent surgery.

• Surgical series (local fascial repair, stoma relocation, mesh repair) show high recurrence rates, especially after suture or relocation alone, and non‑trivial morbidity even with mesh repair.

• No randomized trials compare watchful waiting versus elective repair.

This means that many parastomal hernias remain minimally symptomatic or asymptomatic and never undergo surgery, whereas surgical repair carries substantial recurrence and complication risk.

GRADE – certainty of evidence

• No direct comparative trials; inference based on separate observational cohorts and surgical series.

→ Certainty: **Very low**.

Recommendation

We suggest **watchful waiting** for patients with asymptomatic or minimally symptomatic parastomal hernias. Elective repair should generally be reserved for patients with:

• troublesome appliance problems (leakage, poor fixation),

• pain or discomfort impacting quality of life,

• episodes of obstruction or incarceration, or

• significant skin complications or hygiene issues.

Direction & strength: *For watchful waiting in asymptomatic/minimally symptomatic patients*; **conditional recommendation, very‑low‑certainty evidence**.

---

3. Are there stoma creation techniques without mesh that reduce parastomal hernia incidence?

PICO

Stoma creation **without prophylactic mesh**.

Interventions: transrectus vs pararectal (lateral) route, extraperitoneal vs intraperitoneal track, different aperture sizes.

Comparator: alternative non‑mesh techniques.

Outcome: parastomal hernia incidence.

Summary of evidence

• Classic and more recent studies comparing transrectus versus lateral pararectal routes have not shown a consistent, significant reduction in parastomal hernia incidence with the rectus approach.

• A small randomized pilot trial comparing lateral pararectal versus transrectus loop ileostomy found no significant difference in parastomal hernia rates; the study was underpowered.

• Retrospective series and a systematic review including several hundred patients found no robust advantage of one route over another; overall evidence quality is very low.

• Observational CT‑based cohorts demonstrate that larger aperture size (defect diameter) and greater subcutaneous fat thickness are strongly associated with higher parastomal hernia risk and with symptomatic hernias, but there are no trials randomizing patients to different aperture sizes.

GRADE – certainty of evidence

• All data are observational or from small, underpowered trials; definitions of parastomal hernia vary widely.

→ Certainty: **Very low**.

Recommendation

We cannot recommend any specific **non‑mesh stoma creation technique** (transrectus, lateral pararectal, extraperitoneal) solely for the purpose of reducing parastomal hernia risk. However, based on consistent observational associations:

• Surgeons should avoid unnecessarily large fascia apertures and should pay attention to modifiable risk factors (BMI, waist circumference) when planning stoma creation.

Direction & strength: *No specific technique recommended; cautious suggestion to avoid large apertures and optimize risk factors*; **very‑low‑certainty evidence**.

---

4. Does prophylactic mesh placement during stoma construction reduce hernia occurrence?

PICO

Adults undergoing stoma formation (mainly permanent end colostomy).

Intervention: prophylactic mesh placement at the time of stoma creation (sublay/retromuscular or intraperitoneal IPOM).

Comparator: no mesh.

Outcomes: parastomal hernia incidence (clinical and CT), mesh‑related complications, quality of life, and cost‑effectiveness.

Summary of evidence – synthetic meshes

• Multiple RCTs with synthetic meshes (lightweight polypropylene in sublay/retromuscular position; composite or ePTFE meshes in IPOM position) consistently show a substantial reduction in parastomal hernia incidence versus no mesh, both in open and laparoscopic APR settings.

• Examples include sublay/retromuscular trials and laparoscopic IPOM trials (keyhole and modified Sugarbaker). Across these studies, hernia rates are reduced from ≈25–60% in controls to ≈5–25% in mesh groups, depending on follow‑up and modality (clinical vs CT).

• Meta‑analyses pooling these RCTs report relative risk reductions to about 20–30% of the baseline risk, without a significant increase in overall morbidity, stoma‑related complications, or mesh infections.

• A Markov cost‑effectiveness model using these data suggests that prophylactic synthetic mesh at the time of APR is cost‑effective (and often cost‑saving) for most rectal cancer stages, assuming typical mesh costs and low mesh infection rates.

Summary of evidence – biologic meshes

• A randomized trial using a porcine acellular dermal matrix (sublay) for prophylaxis did not demonstrate a significant reduction in parastomal hernia versus no mesh.

• Small feasibility series with collagen/biologic implants at the stoma site report acceptable short‑term safety but are too small and non‑comparative to demonstrate clear efficacy.

• No convincing evidence shows an advantage of biologic over synthetic meshes in prophylaxis.

GRADE – certainty of evidence

• For **synthetic meshes**: multiple RCTs and a meta‑analysis with consistent benefit, but some heterogeneity in techniques and definitions → **Moderate certainty**.

• For **biologic meshes**: small, often negative or inconclusive datasets → **Low certainty**.

Recommendations

• We recommend prophylactic placement of a **synthetic, lightweight mesh** in a sublay/retromuscular or intraperitoneal IPOM position at the time of elective formation of a permanent end colostomy, provided the patient’s overall risk profile (infection risk, life expectancy, comorbidities) is acceptable.

Direction & strength: *For synthetic prophylactic mesh*; **strong recommendation, moderate‑certainty evidence**.

• We suggest **against** routine use of **biologic meshes** for prophylaxis, as they have not shown a clear reduction in parastomal hernia and are substantially more expensive.

Direction & strength: *Against routine use of biologic mesh*; **conditional recommendation, low‑certainty evidence**.

---

5. Is suture repair an acceptable option for elective parastomal hernia repair?

PICO

Adults with parastomal hernia undergoing **elective** repair.

Intervention: primary suture (fascial) repair.

Comparator: mesh‑based repair (open or laparoscopic) or stoma relocation with or without mesh.

Outcomes: hernia recurrence, complications, reoperations.

Summary of evidence

• Classic and more recent series consistently show very high recurrence rates after primary suture repair:

– Around 70–80% recurrence after the first suture repair.

– Recurrence rates approaching 100% for suture repair of recurrent parastomal hernias.

• Relocation without mesh also has high recurrence rates (≈30–70%), though lower than pure suture repair in some series. However, relocation carries additional risks of midline incisional hernias and other wall defects.

• Systematic reviews pooling multiple series report:

– Recurrence ≈50–80% after suture repair,

– ≈7–17% after mesh repair (open or laparoscopic).

GRADE – certainty of evidence

• All data are from retrospective or prospective case series, without randomization, but findings are strikingly consistent across centres and time.

→ Certainty: **Low** (but effect size is large and consistent).

Recommendation

We recommend **against** pure suture repair of parastomal hernia in elective settings, except in exceptional circumstances where mesh implantation is clearly contraindicated (e.g., gross contamination, severe infection not amenable to staged repair).

Mesh‑based repair (open or laparoscopic) should be the default option when surgery is undertaken.

Direction & strength: *Against pure suture repair*; **strong recommendation, low‑certainty evidence**.

---

6. Is laparoscopic repair equivalent to open repair in elective cases?

PICO

Elective parastomal hernia repair.

Intervention: laparoscopic repair (IPOM).

Comparator: open repair (various mesh techniques or relocation).

Outcomes: recurrence, perioperative morbidity, SSI, length of hospital stay, mortality.

Summary of evidence

• A large registry‑based analysis (NSQIP) comparing laparoscopic and open parastomal hernia repairs, after adjustment for baseline factors, found:

– shorter length of stay with laparoscopy,

– lower overall morbidity and lower SSI rates,

– similar mortality.

Selection bias is likely (healthier patients and less complex hernias preferably offered laparoscopy).

• Smaller comparative series show shorter hospital stays after laparoscopic repair and recurrence rates in the same range or somewhat lower than open mesh repairs, though sample sizes are small.

• There are no randomized trials directly comparing open vs laparoscopic repair.

GRADE – certainty of evidence

• Non‑randomized data, potential for confounding and selection bias.

→ Certainty: **Low**.

Recommendation

We suggest that **laparoscopic repair** be considered for elective parastomal hernia in suitable patients and in centres with appropriate expertise, due to potential advantages in length of stay and wound morbidity. However, available data do not allow us to claim full equivalence or superiority to open repair for all patients and hernia types.

Direction & strength: *For laparoscopic repair in selected elective cases*; **conditional recommendation, low‑certainty evidence**.

---

7. What is the optimal open repair technique?

PICO

Elective open repair of parastomal hernia.

Interventions: different mesh positions (onlay, preperitoneal/sublay/retromuscular, intraperitoneal), stoma relocation, biologic mesh onlay.

Comparator: other open techniques.

Outcomes: recurrence, wound complications, mesh infection, reoperations.

Summary of evidence

• Mesh‑based open repairs generally have considerably lower recurrence rates (≈7–17%) than suture repairs or relocation alone.

• A preperitoneal or retromuscular (sublay) mesh position is conceptually attractive and supported by series reporting recurrence rates around 10% at medium‑ to long‑term follow‑up.

• No direct head‑to‑head RCTs compare onlay vs sublay vs intraperitoneal mesh in open parastomal hernia repair. Systematic reviews do not demonstrate a consistently superior open mesh position, although sublay techniques appear to offer favourable outcomes in experienced hands.

• A study of biologic onlay repair with cross‑linked dermal collagen (Permacol) reported extremely high recurrence rates (≈90%) at long‑term follow‑up, effectively disqualifying this specific approach.

• Hybrid techniques combining laparoscopic adhesiolysis with open mesh placement and stoma revision (e.g., HyPER‑type approaches) report excellent preliminary results (0 recurrences in small series), but sample sizes are very small.

GRADE – certainty of evidence

• For mesh vs no mesh: low‑certainty but consistent benefit.

• For specific open mesh positions (onlay vs sublay vs intraperitoneal): **very‑low‑certainty** evidence.

• For biologic onlay Permacol: low‑certainty but strongly unfavourable outcomes.

Recommendations

• We recommend open **mesh‑based repair** rather than suture or simple relocation alone for elective parastomal hernia.

• Among open techniques, we suggest using a **synthetic mesh in a preperitoneal/retromuscular (sublay) position** when anatomically feasible, as this is supported by reasonable recurrence rates in the available series and by principles applied to other ventral/incisional hernias.

• We recommend **against** biologic onlay repair with cross‑linked dermal collagen (Permacol) for parastomal hernia because of unacceptably high recurrence rates.

Direction & strength:

• *For synthetic mesh open repair (preferably sublay)*; **conditional recommendation, very‑low‑certainty evidence**.

• *Against Permacol onlay*; **strong recommendation, low‑certainty evidence**.

---

8. What is the optimal laparoscopic repair technique?

PICO

Laparoscopic parastomal hernia repair.

Interventions: keyhole IPOM, modified Sugarbaker, sandwich or double‑mesh techniques, funnel‑type IPST meshes.

Comparator: other laparoscopic techniques.

Outcomes: recurrence, mesh complications, bowel obstruction, reoperations.

Summary of evidence

• Systematic reviews and meta‑analyses comparing laparoscopic techniques consistently show lower recurrence rates with **modified Sugarbaker** repairs than with **keyhole** repairs.

– Keyhole techniques have recurrence rates around 25–30%.

– Sugarbaker techniques have recurrence rates around 7–12%.

• A multicentre series of laparoscopic Sugarbaker repairs reported recurrence around 6–7% at medium‑term follow‑up.

• Sandwich techniques and the use of funnel‑type meshes (e.g. IPST) yield very low recurrence rates in small series, but they involve more material and complexity, and long‑term data are limited.

• Mesh‑related complications (e.g. infection, obstruction) are relatively infrequent but present; intraperitoneal PVDF meshes may be associated (in indirect evidence from other ventral hernia populations) with higher rates of adhesions/obstruction than some composite meshes.

GRADE – certainty of evidence

• All data are from non‑randomized series and pooled observational analyses; there is heterogeneity in patient selection and definitions of recurrence.

→ Certainty: **Low**.

Recommendation

We suggest using a **modified Sugarbaker** technique as the preferred laparoscopic method for parastomal hernia repair, based on consistently lower recurrence rates compared to keyhole techniques.

We suggest **against** using a pure keyhole repair as the standard or sole laparoscopic approach because of its higher recurrence rates. Sandwich and 3D funnel techniques appear promising but should currently be considered on a case‑by‑case basis in experienced centres, as evidence is very limited.

Direction & strength: *For Sugarbaker over keyhole*; **conditional recommendation, low‑certainty evidence**.

---

9. Which mesh types are most effective?

PICO

Mesh type in parastomal hernia prophylaxis and repair.

Interventions: synthetic meshes (polypropylene, PVDF, composites), biologic/collagen meshes, funnel/3D meshes (IPST), various coatings/barrier materials.

Comparator: other mesh types or no mesh.

Outcomes: hernia recurrence, mesh infection, chronic pain, bowel obstructions, cost.

Summary of evidence

• In prophylaxis, all RCTs showing benefit have used **synthetic meshes** (lightweight PP or composite/ePTFE IPOM). These significantly reduce parastomal hernia incidence versus no mesh.

• In treatment, case series with synthetic meshes (PP, PVDF, composites) in open or laparoscopic positions report acceptable recurrence rates (often <20%) and low mesh infection rates.

• Biologic meshes and cross‑linked collagen grafts:

– Prophylactic RCT with PADM (Strattice) showed no reduction in hernia versus no mesh.

– Small series of biologic implants suggest feasibility and short‑term safety but no clear long‑term advantage.

– A long‑term series of Permacol onlay repairs demonstrated extremely high recurrence (~90%), indicating poor performance in this application.

• A comparative study of intraperitoneal PVDF versus composite mesh in ventral hernia repair (not specifically parastomal) suggested similar recurrence rates but a higher rate of adhesion‑related obstruction with PVDF; this is indirect but relevant for large IPOM fields around stomas.

GRADE – certainty of evidence

• There are no high‑quality head‑to‑head trials comparing different synthetic meshes in parastomal settings. Most comparisons are between mesh vs no mesh.

• For synthetic vs biologic meshes, data suggest at least no advantage of biologic meshes and occasionally worse durability, but evidence remains indirect and heterogeneous.

→ Certainty:

• **Low–moderate** that synthetic meshes are effective (vs no mesh).

• **Low** that biologic/collagen meshes do **not** offer superior outcomes and may be inferior in some uses (e.g. Permacol onlay).

Recommendations

• We recommend using **synthetic, lightweight meshes** (polypropylene or appropriately designed composite meshes for IPOM) for both prophylaxis and elective repair of parastomal hernia.

• We suggest **against** routine use of **biologic or cross‑linked collagen meshes** (especially as onlay) for prophylaxis or treatment of parastomal hernia, given the lack of demonstrated superiority and in some cases very high recurrence rates and higher costs.

• When considering intraperitoneal PVDF meshes, surgeons should be aware of indirect evidence suggesting a possible higher risk of adhesion‑related bowel obstruction compared to some composite meshes and balance this against other advantages of the material.

Direction & strength:

• *For lightweight synthetic meshes*; **strong recommendation, low–moderate‑certainty evidence (vs no mesh)**.

• *Against routine biologic / cross‑linked collagen meshes*; **conditional (and strong against for Permacol onlay), low‑certainty evidence**.

---

User: Convert this into a GRADE recommendation table.

Assistant:

[Assistant then provided a structured GRADE table with one row per Key Question, summarizing: recommendation, direction & strength, certainty of evidence, and key evidence points.]

---

User: Now prepare a file with all our prompts and your answers from the beginning and save it in English.

Assistant: [This is the current step – generating this English conversation log as a file.]
